# Identity and functions of inorganic and inositol polyphosphates in plants

**DOI:** 10.1111/nph.16129

**Published:** 2019-09-20

**Authors:** Laura Lorenzo‐Orts, Daniel Couto, Michael Hothorn

**Affiliations:** ^1^ Structural Plant Biology Laboratory Department of Botany and Plant Biology University of Geneva 30 Quai E. Ansermet Geneva 1211 Switzerland

**Keywords:** inorganic polyphosphate, inositol pyrophosphates, nutrient signaling, phosphate metabolism, phosphate starvation responses

## Abstract

Inorganic polyphosphates (polyPs) and inositol pyrophosphates (PP‐InsPs) form important stores of inorganic phosphate and can act as energy metabolites and signaling molecules. Here we review our current understanding of polyP and inositol phosphate (InsP) metabolism and physiology in plants. We outline methods for polyP and InsP detection, discuss the known plant enzymes involved in their synthesis and breakdown, and summarize the potential physiological and signaling functions for these enigmatic molecules in plants.

**Table:** 

Contents		
	Summary	637
I.	Introduction	637
II.	Inorganic polyphosphates	638
III.	Inositol pyrophosphates	642
	Acknowledgements	648
	References	648

## I. Introduction

**Figure 1:**
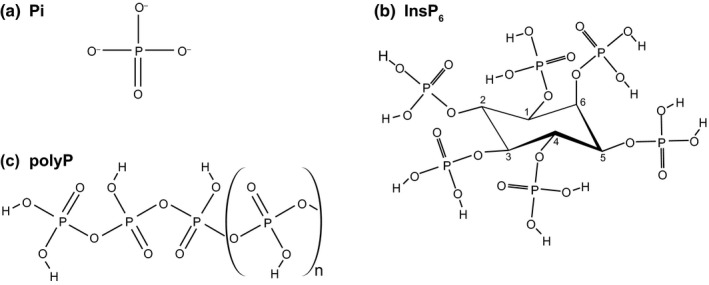
Chemical structure of phosphate‐rich molecules. Representation of (a) phosphate (Pi), (b) inorganic polyphosphate (polyP) and (c) inositol hexakisphosphate (InsP_6_).

Phosphorus is an essential nutrient for all living organisms, representing one of the nine macronutrients present in large quantities in plant tissues. Phosphorus is taken up by plants in the form of inorganic phosphate (Pi; for a list of abbreviations used in this article, see Table 1) (Fig. 1a). Pi is an essential building block for many cellular components, such as nucleic acids and membranes, is a major component of molecules that function as the energy currency of the cell, and is an important signaling molecule. Hence, Pi deficiency can affect a wide range of biological processes, ultimately affecting plant growth and development (Rouached *et al*., 2010).

**Table 1:** Abbreviations list.

Abbreviation	Definition
CHAD	C‐terminal conserved α‐helical domain
CK	Casein kinase
InsP	Inositol polyphosphate
IP6K	Inositol hexakisphosphate kinase
N	Nitrogen
NUDTs	Nudix hydrolase family
P	Phosphorus
PAP	Purple acid phosphatase
PH domain	Pleckstrin homology domain
PHO pathway	Phosphate signal transduction pathway (yeast)
PHR1	PHOSPHATE STARVATION RESPONSE 1
Pi	Inorganic phosphate
PIP	Phosphoinositide
PolyP	Inorganic polyphosphate
PP‐InsP	Inositol pyrophosphate
PPIP5K	Diphosphoinositol pentakisphosphate kinase
PPK	PolyP kinase
PPN	Endopolyphosphatase
pppGpp	Guanosine pentaphosphate
PPX	Exopolyphosphatase
PSR	Phosphate starvation response (plants)
SPX	SYG1/Pho81/XPR1
TTM	Triphosphate tunnel metalloenzyme
VIH	VIP1 HOMOLOG
VTC	Vacuolar transporter chaperone

Plants store Pi in their vacuoles and relocate it to the cytosol when intracellular Pi concentrations are low (Liu *et al*., 2016). Some plant tissues, such as seeds and fruits, can store Pi in the form of phytic acid (inositol hexakisphosphate, InsP_6_; Fig. 1b) (Secco *et al*., 2017). Phytic acid is one of several inositol multiphosphorylated compounds present in plants, and these compounds share the D‐*myo*‐inositol ring‐bearing ester phosphate group at one or more positions. While InsP_6_ may represent a storage form of Pi in plants, higher phosphorylated inositol pyrophosphates act as signaling molecules in plants (Laha *et al*., 2015; Wild *et al*., 2016; Zhu *et al*., 2019) and many other eukaryotes (Azevedo & Saiardi, 2017).

Inorganic polyphosphates (polyPs) exist in many prokaryotes and eukaryotes, and form a major store of Pi, for example, in yeast (Urech *et al*., 1978). PolyPs form linear chains that vary in length from 3 to *c*. 1000 Pi units and are linked by energy‐rich phosphoanhydride bonds (Fig. 1c). Depleting inositol pyrophosphates (PP‐InsPs) in yeast results in a massive decrease in polyP concentrations (Lonetti *et al*., 2011). This suggests that Pi, InsP and polyP metabolism are functionally connected, especially in sessile, soil‐living organisms. However, it is presently unclear whether polyPs exist in plants and whether they contribute to Pi metabolism and storage. Here, we critically review our current knowledge concerning polyPs in plants, and the roles of PP‐InsPs in Pi sensing and cell signaling.

## II. Inorganic polyphosphates

PolyPs were first described in yeast (Liebermann, 1890) and bacteria (Babes, 1895). They also form abiotically; for instance, it has been shown that volcanic activity can generate polyPs from tetraphosphosphorus decoxide (P_5_O_10_) (Yamagata *et al*., 1991). They might therefore have played a role in evolution, when Pi was mostly found as part of prebiotic phosphorous minerals. The presence of polyPs in both prokaryotes and eukaryotes suggests an ancient origin for this polymer.

### II..1 Localization and functions of inorganic polyphosphates

**Figure 2:**
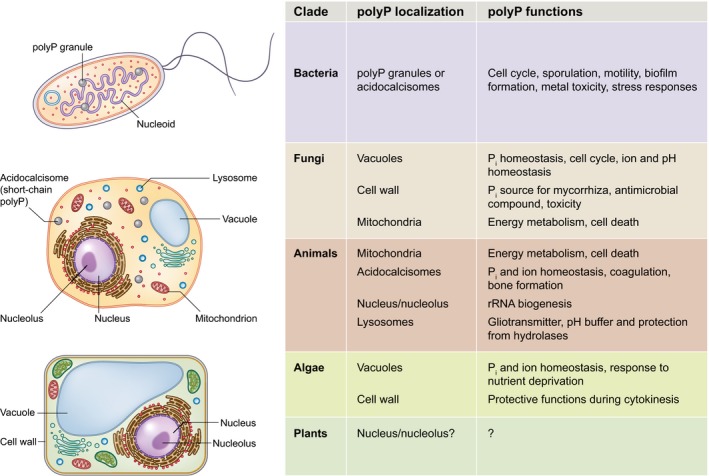
Localizations and functions of polyPs in different organisms. Subcellular localization of inorganic polyphosphates (polyPs) is shown schematically on the left. A table summarizing the functions reported in the different cellular compartments is shown on the right.

Many functions have been attributed to polyPs in various organisms (Fig. 2). In bacteria, polyPs accumulate within granules in the nucleoid region (Racki *et al*., 2017) and can regulate various cellular processes, including growth, sporulation, response to nutrient deprivation, virulence, cell cycle and metal toxicity (Xie & Jakob, 2019). PolyPs also act as primordial chaperones in bacteria, binding to unfolded proteins and promoting their refolding (Gray *et al*., 2014). Furthermore, they are able to associate with calcium ions (Ca^2+^) and polyhydroxybutyrate in the bacterial membrane, forming DNA entry channels that are involved in bacterial competence (Castuma *et al*., 1995).

In yeast, polyPs are located mainly in the vacuole, although a minor fraction (< 10%) can be found in mitochondria (Pestov *et al*., 2004), where they control energy metabolism and cell death (Abramov *et al*., 2007). In vacuoles, they act as a major storage form of Pi, energy and divalent cations, accumulating when intracellular Pi concentrations are high. Under Pi starvation, yeast cells can remobilize Pi from polyP (Shirahama *et al*., 1996). The vacuolar polyP store thus allows yeast cells to maintain Pi, ion (Rosenfeld *et al*., 2010) and pH (Eskes *et al*., 2018) homeostasis. PolyPs control the cell cycle by providing the Pi required for nucleotide synthesis during DNA replication (Bru *et al*., 2016). Outside the vacuole, polyPs have been observed in the fungal cell wall, where they are thought to play a role in providing Pi in symbiotic interactions (Chiu & Paszkowski, 2019) and protecting fungal cells from pathogens and toxic compounds (Werner *et al*., 2007b).

In the amoeba *Dictyostelium discoideum*, polyPs accumulate in acidocalcisomes (Zhang *et al*., 2005). Acidocalcisomes are acidic vacuoles, which contain Ca^2+^ and polyP and thus play a role in several processes, including calcium signaling and autophagy (Docampo *et al*., 1995). In trypanosomes, polyPs also accumulate in acidocalcisomes, although they have been detected in the nucleolus and the glycosome as well (Negreiros *et al*., 2018).

Algae accumulate polyPs in vacuoles with similar properties to acidocalcisomes (Ruiz *et al*., 2001; Aksoy *et al*., 2014), which maintain Pi and ion homeostasis. PolyPs also play a role in nutrient deprivation; mutants lacking a component of the polyP polymerase complex are more susceptible to sulfur, phosphorus and nitrogen starvation (Aksoy *et al*., 2014).

In humans, polyPs have been found in different subcellular compartments, including acidocalcisomes (Ruiz *et al*., 2004), lysosomes (Pisoni & Lindley, 1992) and the nucleolus (Jimenez‐Nuñez *et al*., 2012). PolyPs play a role in diverse biological functions in humans, for instance in the regulation of rRNA biogenesis (Jimenez‐Nuñez *et al*., 2012; Azevedo *et al*., 2015), coagulation (Müller *et al*., 2009), and bone formation (Hacchou *et al*., 2007).

PolyPs have been demonstrated to be covalently linked to lysine residues in yeast and human proteins involved in ribosome biogenesis (Azevedo *et al*., 2015; Bentley‐DeSousa *et al*., 2018). In yeast, protein polyphosphorylation is genetically linked to inositol pyrophosphate metabolism (Azevedo *et al*., 2015). Whether lysine polyphosphorylation represents an important post‐translational modification in plants requires further investigation.

### II..2 Enzymes metabolizing inorganic polyphosphates

Specific polyP‐metabolizing enzymes have been identified in bacteria and lower eukaryotes, as well as in humans, yet they have not been functionally characterized in multicellular organisms. Bacterial polyP kinases 1 and 2 (PPK1/2) catalyze the synthesis of polyP from ATP and/or GTP (Ahn & Kornberg, 1990; Zhang *et al*., 2002). PPK1 can also regenerate ATP from polyP (Ahn & Kornberg, 1990). In yeast, polyP synthesis is carried out by the vacuolar transporter chaperone (VTC) complex, which contains several subunits (Hothorn *et al*., 2009). The catalytic subunit Vtc4 contains a tunnel‐shaped (TRIPHOSPHATE TUNNEL METALLOENZYME, TTM) catalytic domain, which catalyzes the synthesis of polyP from ATP. The growing polymer is translocated into the vacuole by means of a trans‐membrane domain, to which several subunits contribute (Hothorn *et al*., 2009; Gerasimaitė *et al*., 2014). VTC is conserved in all unicellular eukaryotes, including *Chlamydomonas* (Aksoy *et al*., 2014) and trypanosomes (Lander *et al*., 2013). *Dictyostelium discoideum* can produce polyP via a protein homolog of the bacterial PPK1 (Zhang *et al*., 2005), and an actin‐related protein named DdPPK2, which forms filaments concurrently with the synthesis of polyP (Gómez‐García & Kornberg, 2004).

Specific inorganic polyphosphatases have been discovered in prokaryotes and eukaryotes. In bacteria, long‐chain polyPs can be sequentially hydrolyzed by exopolyphosphatase 1 (PPX1; Akiyama *et al*., 1993). PPX1 belongs to the same protein superfamily as actin, HSP70 chaperones and sugar kinases, and hydrolyzes both polyP and the alarmone guanosine pentaphosphate (pppGpp; Kuroda *et al*., 1999). The short‐chain inorganic polyphosphatase ygiF from *Escherichia coli* hydrolyzes tripolyphosphate into pyrophosphate and Pi (Kohn *et al*., 2012; Martinez *et al*., 2015). ygiF contains an N‐terminal TTM domain with structural homology to yeast Vtc4 and a C‐terminal conserved α‐helical domain (CHAD). In yeast, PPX1 belongs to the DHH phosphatase family and hydrolyzes the terminal Pi from short‐chain polyPs (Wurst & Kornberg, 1994). Human H‐prune belongs to the same protein family as yeast PPX1 and can hydrolize polyP (preferentially short‐length polyP) as well as nucleoside tetraphosphates (Tammenkoski *et al*., 2008). Members of the nudix hydrolase family (NUDTs), which have been reported to cleave InsPs, can also cleave polyPs *(Lonetti et al.,* 2011). In addition, the human purple acid phosphatase ACP5 is able to cleave polyP, with a substrate preference for short‐chain polyPs (Harada *et al.,* 2013).

There are also endopolyphosphatases that release Pi from an internal or terminal position in a polyP chain. PPN1 localizes to the yeast cytosol and belongs to the calcineurin‐like phosphatase family (Kumble & Kornberg, 1995), while PPN2 is a metallophosphatase in the vacuolar lumen (Gerasimaitė & Mayer, 2017)

### II..3 Inorganic polyphosphate metabolizing enzymes and binding proteins in plants

No *bona fide* PPK1, PPK2 or Vtc4 orthologs have been identified in higher eukaryotes, including plants, and polyP synthesis is therefore poorly understood in these organisms (Kumble & Kornberg, 1995; Pavlov *et al*., 2010). However, several conserved proteins with significant homology to bacterial or animal inorganic polyphosphatases are present in plant genomes, including the model plant *Arabidopsis thaliana* (Table 2).

**Table 2:** *Arabidopsis thaliana* orthologs of polyP polymerases and phosphatases from bacteria, yeast and *Dictyostelium discoideum.*

PolyP polymerases			Arabidopsis protein homologs		
Organism	Enzyme	Protein domain	Seq.	Secondary structure	Example
Bacteria	PPK1	Phospholipase D	–	Phospholipase D domain: *c*. 40	At1g55180
	PPK2	Thymidine kinase	–	Thimidine kinase: 2	At3g07800 (TK1A)
Yeast	Vtc4	VTC	–	CYTH/TTM domain: 3	At2g11890 (TTM3)
*Dictyostelium*	PPK1	Phospholipase D	–	Phospholipase D domain: *c*. 40	At1g55180
	DdPPK2	Actin‐related protein 1	–	–	–

Polyphosphatases			Arabidopsis protein homologs		
Bacteria	PPX1	PPX (actin, HSP70, sugar kinases)	1	PPX domain: 1	At1g09195
	ygiF	CYTH	–	CYTH/TTM domain: 3	At2g11890 (TTM3)
Yeast	PPX1	DHH phosphoesterases	–	DHH domain: 5; DHHA1 domain: 7	At1g53345
	PPN1	Calcineurin‐like phosphoesterases	–	Calcineurin‐like phosphoesterases: 190	At3g03305
	PPN2	Metallophosphatase	–	–	–
Human	H‐prune	DHH phosphoesterases	–	DHH domain: 5; DHHA1 domain: 7	At1g53345
	DIPP1/2/3	Nudix hydrolases	2	Nudix hydrolase domain: *c*. 100	At4g25434 (NUDX10)
	ACP5	Purple acid phosphatases	*c*. 10	Purple acid phosphatases: 16	At2g01880 (PAP7)

The number of proteins with primary and secondary structure homology to inorganic polyphosphate (polyP)‐metabolizing enzymes is listed. Putative polyP‐metabolizing enzymes from Arabidopsis were identified by sequence homology using a protein–protein BLAST search (Altschul *et al*., 1990), or by similarities in domain architecture using interpro (Mitchell *et al*., 2019). Note that the biochemical activities for many of the Arabidopsis proteins listed have not been experimentally assessed.

AtTTM3 shares structural homology to both the catalytic domain of the polyP polymerase Vtc4 (Hothorn *et al*., 2009) and the *E. coli* short‐chain polyP phosphatase ygiF (Martinez *et al*., 2015). Enzymatic and structural characterization of AtTTM3, which is conserved in the entire plant lineage (Lorenzo‐Orts *et al*., 2019b), revealed that it is a *bona fide* ortholog of bacterial ygiF, hydrolyzing short‐chain polyPs using a conserved catalytic mechanism based on two metal ion centers (Moeder *et al*., 2013; Martinez *et al*., 2015). AtTTM3 is a broadly expressed, soluble protein localized in the cytosol and nucleus (Lorenzo‐Orts *et al*., 2019b). Genetic characterization of AtTTM3 initially revealed a function in root growth (Moeder *et al*., 2013). Subsequent analyses, however, revealed that TTM3 is transcribed into and translated from a bicistronic mRNA, encoding both TTM3 and the cell division protein 26 (CDC26; Lorenzo‐Orts *et al*., 2019b). While CDC26 is essential for Arabidopsis embryo development and regulates plant growth, AtTTM3 seems to be dispensable under favorable growth conditions (Lorenzo‐Orts *et al*., 2019b). While the physiological roles of AtTTM3 remain unclear, it is of note that functional connections between polyPs and cell cycle regulation are well established in other prokaryotes and eukaryotes (Bru *et al*., 2016; Racki *et al*., 2017). There are at least two additional TTM proteins in plants (AtTTM1 and AtTTM2), where the TTM domain is located at the C‐terminus of a uridine kinase domain (Ung *et al*., 2014, 2017). Both enzymes have been reported to have pyrophosphatase activity, but it is not known which domain harbors this activity (Ung *et al*., 2014, 2017).

A putative yet thus far uncharacterized ortholog of bacterial exopolyphosphatase is present in the Arabidopsis genome as a single‐copy gene (*At1g09195*) and appears to be conserved among many plant species. PPX enzymes have been reported to cleave both polyP and pppGpp (Kuroda *et al*., 1999). Enzymes catalyzing the synthesis and breakdown of pppGpp in plants (van der Biezen *et al*., 2000) have been shown to function in the chloroplast, where pppGpp accumulates under stress conditions (Chen *et al*., 2014). It has been speculated that PPX could cleave pppGpp, regulating stress responses (Boniecka *et al*., 2017). However, the enzymatic properties and physiological functions of AtPPX require further investigation.

In Arabidopsis, there are 28 putative NUDTs (Yoshimura & Shigeoka, 2015). Human NUDTs have been shown to hydrolyze polyPs, InsPs and other nucleoside polyphosphates (Lonetti *et al*., 2011). Plant NUDTs act on a large variety of substrates, including ADP‐ribose (Ogawa *et al*., 2005), nucleotides modified by reactive oxygen species (Jemth *et al*., 2019), and the alarmone pppGpp (Ito *et al*., 2012). The large number of NUDTs in Arabidopsis and the diverse substrate specificity of NUDT family members render their contribution to polyP metabolism difficult to assess. This is even more true for plant purple acid phosphatases (PAPs), metalloenzymes which hydrolyze a wide range of Pi esters and anhydrides. In Arabidopsis, there are 29 predicted PAPs, although only 28 of them have been shown to be at least transcribed (Tran *et al*., 2010). Among them, AtPAP15 was reported to have phytase activity (Zhang *et al*., 2008), but polyPs were not tested as potential substrates.

Recently, we have characterized CHAD, present for example in the bacterial short‐chain polyphosphatase ygiF, as a specific polyP‐binding module (Lorenzo‐Orts *et al*., 2019a). CHADs can be found in the three domains of life and co‐localize with polyP granules in bacteria (Tumlirsch & Jendrossek, 2017), yet they do not exhibit enzymatic activity towards polyPs (Martinez *et al*., 2015). Interestingly, the plant *Ricinus communis* has a CHAD‐containing protein (Lorenzo‐Orts *et al*., 2019a). Its high structural and sequence similarity with bacterial CHAD domains and its singularity in the plant kingdom suggests that RcCHAD might have been acquired by *Ricinus* via horizontal gene transfer from a soil‐living bacterium. RcCHAD is expressed in *Ricinus* and binds polyPs with low micromolar affinity and high selectivity (Lorenzo‐Orts *et al*., 2019a). As with other genes acquired by horizontal gene transfer, RcCHAD may have evolved a new cellular function or may bind polyP as in bacteria.

### II..4 Inorganic polyphosphate detection in cells and tissues

Early studies report the detection of polyPs in different algae and mosses and in higher plants, such as spinach (leaves) and cotton (seeds) (Keck & Stich, 1957; Miyachi, 1961; Tewari & Singh, 1964). In line with this, electron‐dense granules inside and outside plant vacuoles have been interpreted as polyP bodies in transmission electron microscopy (TEM) sections (Mamun *et al*., 2005). Here, we will briefly review the different methods available for the identification and quantification polyP stores in cells and tissues to put these findings in context.

The yeast polyphosphatase PPX1, which releases the terminal Pi from polyP molecules, has been used to estimate polyP concentrations in cell extracts. The product of this reaction can be estimated with malachite green, which allows Pi concentrations to be quantified via a simple colorimetric reaction (Bru *et al*., 2017). This assay may be sensitive to contamination by free Pi or other Pi‐containing metabolites. Alternatively, the reverse reaction of PPK1 causes conversion of ADP to ATP, which can be quantified via luciferase‐based assays (Ault‐Riché *et al*., 1998).

Nuclear magnetic resonance (NMR) spectroscopy has long been used to detect the presence of polyP in cells by detecting the magnetic field of ^31^P atoms (Klein *et al*., 1988; Bental *et al*., 1991). This method has been used in plants, but polyPs were not detected (Pratt *et al*., 2009). The sensitivity and resolution of this method are limited, however, hindering the detection of metabolites present at low concentrations (Gowda & Raftery, 2015).

Using TEM, polyP bodies appear as electron‐dense granules, and they have been observed in palm seeds and rice anthers (DeMason & Stillman, 1986; Mamun *et al*., 2005). One way to determine the composition of these granules is by combining electron microscopy with energy‐dispersive X‐ray microanalysis. This technique allows for the detection of atoms based on their emission spectra upon X‐ray excitation, and it has been used to analyze the composition of polyP granules in bacteria (Alvarez & Jerez, 2004) and plants (DeMason & Stillman, 1986; Otegui *et al*., 2002).

Raman microscopy has been successfully adopted for the detection of polyP in various organisms, including bacteria (Majed *et al*., 2009) and microalgae (Moudříková *et al*., 2017). In plants, phytic acid (InsP_6_) could be detected in the aleurone and endosperm tissue of wheat grains using this method, but no specific signal was reported for polyP (Kolozsvari *et al*., 2015). Although this technique offers a high specificity, Kolozsvari *et al*. suggest that the sensitivity of the method needs to be further improved.

Besides specifically staining nucleic acids, 4′‐6‐diamidino‐2‐phenylindole (DAPI) has been extensively used to detect polyP in cells. When bound to DNA, DAPI emits light at a wavelength of 461 nm. DAPI can also bind to RNA (emitting light at *c*. 500 nm) (Kapuscinski, 1990) and to Pi‐rich compounds, including inositol polyphosphates and polyP (emitting light at *c*. 550 nm) (Kolozsvari *et al*., 2014). Thus, DAPI cannot distinguish between inorganic and inositol polyphosphate molecules (Kolozsvari *et al*., 2014).

Since DAPI can bind to different Pi‐containing compounds, novel fluorescent dyes have been reported that bind polyPs with higher specificity. JC‐D7 and JC‐D8 bind polyP and heparin but do not bind to other Pi‐containing molecules such as nucleic acids, nucleotides, or sodium phosphate (Angelova *et al*., 2014). However, both JC‐D7 and JC‐D8 dyes have lower affinities for polyP than for DAPI, and binding to inositol polyphosphates was not assessed (Angelova *et al*., 2014). We recently used the JC‐D7 dye to stain polyP in plant cells (Zhu *et al*., 2019). While plant cells expressing the *E. coli* polyP kinase PPK1 revealed the presence of JC‐D7‐stained polyP granules in the cytosol, no polyP granules could be detected in wild‐type Arabidopsis plants, in the *ttm3‐1* mutant (see section 2.3; Lorenzo‐Orts *et al*., 2019b) or in other monocotyledonous and dicotyledonous plants. Specific staining could only be shown in *Chlamydomonas*, where the VTC complex generates a polyP store in acidocalcisomes (Aksoy *et al*., 2014). These findings suggest that polyPs are either absent in higher plants, do not accumulate to high concentrations, may be present only in specific cell types or organs, or are only produced in response to certain environmental stimuli. Our observations cannot explain the fact that the inorganic polyphosphatase TTM3 is a broadly expressed enzyme in Arabidopsis (Moeder *et al*., 2013; Lorenzo‐Orts *et al*., 2019b). It is worth noting that cytosolic accumulation of PPK1‐generated polyP granules in plants is toxic (Zhu *et al*., 2019), as previously reported in yeast (Gerasimaitė *et al*., 2014).

The polyP‐binding domain (PPXc) of the *E. coli* polyphosphatase PPX has been used to detect polyPs in cells via immunofluorescence. Initially reported in yeast (Saito *et al*., 2005), PPXc has been further used to specifically detect polyP in cells from different organisms, including humans (Jimenez‐Nuñez *et al*., 2012; Moreno‐Sanchez *et al*., 2012) and trypanosomatids (Negreiros *et al*., 2018). We have generated a fluorescent protein fusion of PPXc, which when expressed in isolation localizes to the cytoplasm and nucleus in Arabidopsis and *Nicotiana benthamiana (Zhu et al.,* 2019). The fusion protein, however, localizes to JC‐D7‐stained polyP granules in PPK1‐expressing transgenic lines, suggesting that it can be used to label polyP pools in higher plants (Zhu *et al*., 2019).

A fluorescent protein‐tagged version of the polyP‐binding protein RcCHAD in Arabidopsis and *N. benthamiana* showed a specific localization to the nucleus and nucleolus *(Lorenzo‐Orts et al.,* 2019a). Co‐expression with *E. coli* PPK1 relocalized RcCHAD to PPK1‐generated cytoplasmic polyP granules, suggesting that CHAD domains may be suitable for polyP detection in plant cells and that there may be a nuclear/nucleolar polyP store in higher plants (Lorenzo‐Orts *et al*., 2019a). Further experiments are required to substantiate this finding, but it is of note that nucleolar polyP stores have been reported in human cells (Jimenez‐Nuñez *et al*., 2012) and trypanosomes (Negreiros *et al*., 2018). Furthermore, nucleolar proteins implicated in ribosome biogenesis in yeast and humans are known to be targets of polyphosphorylation (Azevedo *et al*., 2015; Bentley‐DeSousa *et al*., 2018), and it is therefore possible that RcCHAD may bind to nucleolar proteins carrying this post‐translational modification.

### II..5 Inorganic polyphosphates in symbiotic interactions

Approx. 90% of all plant species possess roots that are associated with fungi, forming symbioses known as mycorrhizas. Plant roots provide carbohydrates to the fungus, while the fungus offers mineral nutrients (mainly P and N) to the plant (Bonfante & Genre, 2010). Interestingly, mycorrhizal fungi can accumulate *c*. 60% of the total Pi content in the form of polyP (Hijikata *et al*., 2010). Hence, most of the Pi transported to plant cells is derived from fungal polyP pools (Chiu & Paszkowski, 2019). During colonization, the fungal polyP content increases, with short‐chain polyP being more abundant (Ohtomo & Saito, 2005). How Pi and/or polyPs are transported into the apoplast is currently unknown. One possibility is that the fungal VTC complex (Hothorn *et al*., 2009) can polymerize polyP at the plasma membrane into the apoplast, where the polymer may be hydrolyzed to Pi by polyphosphatases and taken up by the root via mycorrhiza‐inducible Pi transporters (Chiu & Paszkowski, 2019).

#### Perspectives

PolyPs have been reported in many prokaryotic and eukaryotic organisms. In the green lineage, the presence of significant polyP stores has been firmly established in algae (Werner *et al*., 2007a; Aksoy *et al*., 2014), while the microscopy and biochemical extraction methods used in the early studies on higher plants are now known to lack specificity towards polyPs (Keck & Stich, 1957; Miyachi, 1961; Tewari & Singh, 1964). The identity of the polyP bodies reported in plant TEM sections has likewise not been validated by other methods (DeMason & Stillman, 1986; Mamun *et al*., 2005). We have recently used a polyP‐specific dye and a polyP‐binding protein to detect polyPs in plants in a genetically validated manner (Zhu *et al*., 2019). Our experiments suggest that higher plants may not contain large polyP stores in organized vacuoles/acidocalcisomes, at least in the species, tissues and experimental conditions tested (Zhu *et al*., 2019). We found, however, that two polyP‐binding domains (PPXc and RcCHAD) localize to the nucleus and nucleolus in plant cells (Lorenzo‐Orts *et al*., 2019a; Zhu *et al*., 2019). These findings certainly require further validation, but they nevertheless indicate that polyP stores may exist in these compartments. We speculate that lower concentrations of polyPs may not be detectable by fluorescent dyes (Angelova *et al*., 2014; Kolozsvari *et al*., 2014), by ^31^P NMR (Pratt *et al*., 2009) or by Raman spectroscopy (Kolozsvari *et al*., 2015). However, they might be detectable using high‐affinity polyP‐binding domains, which have previously been used to stain polyPs in different compartments in animal cells (Jimenez‐Nuñez *et al*., 2012). Taken together, these findings indicate that the presence of polyPs in higher plants has not been firmly established. However, a number of biochemically validated or putative polyP‐binding proteins and polyP‐metabolizing enzymes are present in plants, some of which are evolutionarily conserved (see section 2.3). This raises the question of what the metabolic and physiological functions of these enzymes might be, and whether or not they act on polyP substrates *in vivo*. Further improvement of the polyP detection methods and in‐depth characterization of known and novel polyP‐metabolizing enzymes may yield a final answer to the question of whether polyPs exist in plants.

## III. Inositol pyrophosphates

### III..1 Inositol pyrophosphates: metabolism

**Figure 3:**
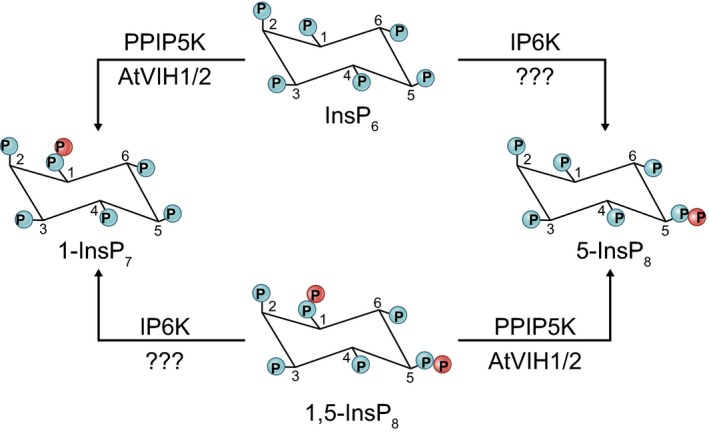
Synthesis of inositol pyrophosphates. Inositol hexakisphosphate (InsP_6_) is converted to inositol pyrophosphate (InsP_7_) upon phosphorylation at positions 1 or 5 by diphosphoinositol pentakisphosphate kinase (PPIP5K) or inositol hexakisphosphate kinase (IP6K) enzymes, respectively. Each reaction product can be further phosphorylated into InsP_8_ by the reciprocal enzyme. The identity of an IP6K‐like enzyme in plants remains unknown.

Inositol polyphosphates (InsPs) are another class of Pi‐rich metabolites present in all eukaryotes; they are versatile Pi storage and signaling molecules that control a wide range of biological processes (Azevedo & Saiardi, 2017; Shears, 2018). InsPs are based on a *myo*‐inositol ring that may be sequentially and reversibly phosphorylated at each of its six carbon positions, giving rise to six different InsP species, termed InsP_1_ to InsP_6_ according to the number of phosphate groups they possess (Fig. 3). The different InsP derivatives have distinct properties, and they can be distinguished by a prefix that indicates the position of the phosphate on the ring (e.g. 1,4,5‐InsP_3_ represents *myo*‐inositol 1,4,5‐trisphosphate; Fig. 3).

InsPs may be further phosphorylated on existing phosphate groups to give rise to inositol pyrophosphates (PP‐InsPs) containing one or more high‐energy pyrophosphate groups. InsPs and PP‐InsPs are present in all eukaryotes, including plants, at varying concentrations depending on the organism and cell type. Some plants accumulate high concentrations of InsP_6_, especially in seeds, where it is thought to act as a Pi storage molecule (Raboy, 2003). By contrast, PP‐InsPs typically make up only a small fraction of the total InsP pool, which poses an inherent problem for accurate detection of these molecules *in vivo* (Desai *et al*., 2014; Laha *et al*., 2015).

The most common PP‐InsPs arise from conversion of InsP_6_ to 1‐InsP_7_ or 5‐InsP_7_, in reactions catalyzed by two distinct classes of enzyme: diphosphoinositol pentakisphosphate kinase (PPIP5K, known as Vip1 in budding yeast) and inositol hexakisphosphate kinase (IP6K, known as Kcs1 in budding yeast), respectively (Shears & Wang, 2019) (Fig. 3). These enzymes can then act on each other's products to give rise to 1,5‐InsP_8_, a signaling molecule that is present at low concentrations in cells. How PPIP5K and IP6K enzymes are regulated in order to modulate PP‐InsP concentrations remains obscure. Since both enzyme families require relatively high concentrations of ATP (KD (equilibrium dissociation constant) values are in the low millimolar range) for PP‐InsP synthesis (Gu *et al*., 2017b; Zhu *et al*., 2019), it is possible that their activities may respond to changes in cellular ATP concentrations. Moreover, the PPIP5Ks are bifunctional enzymes that harbor both a kinase and a phosphatase domain that can add or remove a pyrophosphate group, respectively (Wang *et al*., 2011; Zhu *et al*., 2019). How the functions of these two domains are integrated to regulate PP‐InsPs concentrations has not been established at the mechanistic level.

Yeast has one copy of each enzyme; single or double deletion strains exhibit pleiotropic defects in cell division, Pi metabolism, telomere maintenance and vesicle trafficking (Wilson *et al*., 2013). Mammals possess three IP6K and two PPIP5K isoforms. Genetic deletion of single isoforms from each enzyme family in mice or human cell lines affects multiple processes, including cell growth, apoptosis, glycolysis, insulin production and even hearing loss (Chakraborty, 2018). In the green lineage, PPIP5K homologs have been characterized in Arabidopsis and in *Chlamydomonas reinhardtii* (Laha *et al*., 2015; Couso *et al*., 2016). However, no sequence‐related IP6K homolog could be found in algae or plants, even though the detection of InsP_8_ (Desai *et al*., 2014; Laha *et al*., 2015) suggests the presence of a functional IP6K enzyme.

### III..2 Mechanisms of inositol pyrophosphate signaling

**Figure 4:**
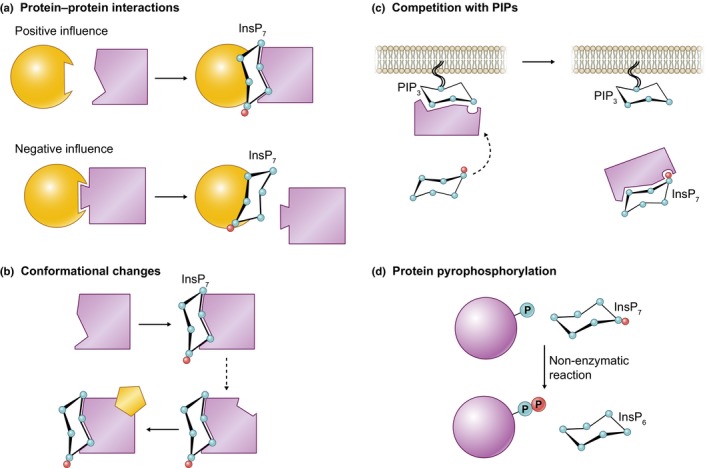
Possible signaling mechanisms of inositol pyrophosphates. (a) Inositol pyrophosphates (PP‐InsPs) may positively or negatively influence protein–protein interactions by creating new or blocking existing interaction surfaces, respectively. (b) Upon PP‐InsP binding, structural readjustments may occur that facilitate subsequent binding to other molecules or proteins. (c) PIPs present in cell membranes act as anchor points for proteins; PP‐InsPs may compete for the same PIP binding site, dislodging the protein from the membrane. (d) PP‐InsPs may transfer non‐enzymatically one of their phosphoryl groups into a phospho‐serine residue that has been previously phosphorylated by a kinase.

Different mechanisms by which PP‐InsPs control cell signaling have been established. The binding of PP‐InsPs to target proteins may influence protein–protein interactions and/or allosterically regulate their activity, and they may compete for the same binding site as membrane‐localized phosphoinositides (PIPs). In addition, PP‐InsPs may directly pyrophosphorylate proteins as a signaling‐competent post‐translation modification. Each of these cases is discussed in the paragraphs that follow, and they are illustrated in Fig. 4.

#### Protein–protein interactions

Signaling proteins are typically part of dynamic complexes that determine their signaling competence, activation state and subcellular localization. Post‐translational modifications or binding of small molecules such as InsPs or PP‐InsPs can greatly influence the interactions between two or more proteins. InsP_4‐6_ were initially shown to promote activation of mammalian casein kinase 2 (CK2; Solyakov *et al*., 2004), a ubiquitous kinase with multiple targets. InsP binding to CK2 interferes with the binding of its negative regulator Nopp140 (Lee *et al*., 2013). Importantly, the PP‐InsP 5‐InsP_7_ binds CK2 more tightly than InsP_6_ and is thus more potent in its activation of CK2 to promote p53‐dependent cell death in human cancer cells (Rao *et al*., 2014). Since 5‐InsP_7_ binds to the substrate‐binding site of CK2, it is tempting to speculate as to whether PP‐InsPs can modulate the substrate specificity of protein kinases. CK2 but not Nopp140 is conserved in all eukaryotes, including plants. Whether plant CK2 isoforms can bind PP‐InsPs to regulate interaction with their substrates is presently unknown.

As well as blocking protein–protein interactions, InsPs and PP‐InsPs may also promote them. This holds true, for example, for PP‐InsP sensing SPX (SYG1/Pho81/XPR1) domains interacting with PHOSPHATE STARVATION RESPONSE (PHR) transcription factors (Wild *et al*., 2016). SPX domains are found in all eukaryotes, where they are located at the N‐terminus of transporters or enzymes, or, in the case of plants, exist as ‘stand‐alone’ modules. These stand‐alone SPX proteins interact with PHR transcription factors only in the presence of InsPs/PP‐InsPs (Wild *et al*., 2016). SPX‐PHR complexes preferably bind highly phosphorylated InsPs, with the PP‐InsP 5‐InsP_7_ binding 10 times more tightly compared to InsP_6_ (Wild *et al*., 2016). When in complex with PHR, SPX proteins prevent the transcription factor from binding DNA and from activating transcription of Pi‐starvation genes (Puga *et al*., 2014; Qi *et al*., 2017). How different PP‐InsPs may affect SPX‐PHR complexes, and which one is preferred *in vivo*, has not yet been determined (see the 'PP‐InsPs and nutrient sensing' section).

#### Allosteric regulation

SPX domains and PP‐InsPs also play a regulatory role in the yeast phosphate signal transduction (PHO) pathway, specifically in the regulation of the Pho85–Pho80 CDK‐cyclin complex. Here, the SPX domain‐containing CDK inhibitor Pho81 constitutively associates with Pho85–Pho80, but it inhibits Pho80–Pho85 only in the presence of PP‐InsPs (Lee *et al*., 2007). Pho81 interacts primarily with Pho80 at a site that is distant from both the substrate docking site on Pho80 and from the Pho85 kinase active site (Huang *et al*., 2007). PP‐InsP binding to the Pho81–Pho85–Pho80 complex is thus likely to induce conformational changes in Pho81 that allow it to block either substrate binding to Pho80 or access to the active site on Pho85. Intriguingly, PP‐InsP binding and Pho85–Pho80 inhibition are independent of the Pho81 SPX domain (Lee *et al*., 2008), whose function in this pathway has not yet been fully investigated.

PolyP synthesis by the yeast VTC complex is activated by several PP‐InsP isoforms (InsP_6_ has a minor effect) that bind to the SPX domains of Vtc2–5 (Wild *et al*., 2016; Gerasimaite *et al*., 2017). The exact underlying mechanism requires further investigation, but it appears that the allosteric PP‐InsP–SPX interaction induces conformational changes in VTC proteins, facilitating ATP hydrolysis and polyP synthesis. Notably, alanine substitutions on key PP‐InsP binding lysines from Vtc3 and Vtc4 result in auto‐active variants that are capable of synthesizing polyP in the absence of PP‐InsPs (Wild *et al*., 2016). Structural analysis of such mutants may be the key to understanding how PP‐InsPs stimulate the VTC complex.

#### Competition with membrane‐bound phosphoinositides

Phosphoinositides (PIPs) are ubiquitous in eukaryote membranes, where they do not have a structural role, but rather regulate membrane–cytosol communication (for a review, see Schink *et al*., 2016). Similarly to soluble InsPs, the inositol ring on PIPs can be reversibly phosphorylated at different positions to yield distinct species with specific membrane localizations. Different PIP species can influence cell signaling by providing an anchoring point for both soluble proteins and cytoplasmic domains of membrane proteins. Pleckstrin homology (PH) domains are responsible for the translocation and membrane anchoring of several proteins by selective binding to PIPs (Lemmon, 2007). Snyder and collaborators demonstrated that InsP_7_ could specifically compete with 3,4,5‐PIP_3_ for binding to the PH domain of several mammalian and *Dictyostelium* proteins (Luo *et al*., 2003). A physiological role was suggested for this mechanism, where 5‐InsP_7_ inhibits activation of the PH domain‐containing Akt kinase by blocking its translocation to the membrane during insulin signaling (Chakraborty *et al*., 2010). Remarkably, 5‐InsP_7_ and InsP_6_ are more efficient at competing with PIP3 for the PH domain of human PBD1 than 1‐InsP_7_ and 1,5‐InsP_8_ *in vitro* (Gokhale *et al*., 2013), suggesting a high degree of specificity. Since PH domains are abundant and diverse and occur fused to a variety of proteins in plants, it is important to assess their affinities for PIPs, InsPs and PP‐InsPs. No plant PH domain has, to the best of our knowledge, been tested for its ability to bind PP‐InsPs.

Other protein domains can mediate binding to PIPs, and thus PIP vs PP‐InsP competition might not be an exclusive feature of PH domains. For example, the synaptotagmin‐1 (Syt1) C2AB domains bind to 4,5‐PIP_2_ clusters during synaptic vesicle fusion (Park *et al*., 2015). In turn, 5‐InsP_7_ inhibits synaptic vesicle fusion by binding to the C2AB domains of Syt1 (Lee *et al*., 2016), but whether this is a clear case of competition for the same binding site remains to be shown. Competition between PP‐InsPs and PIPs may be a mechanism for the rapid adjustment of the localization of proteins shuttling between the cytosol and membrane. Determining how widespread this mechanism is and how it is influenced by local PP‐InsP pools represents an interesting challenge for the future.

#### Non‐enzymatic protein pyrophosphorylation

PP‐InsPs have the ability to non‐enzymatically pyrophosphorylate proteins by transferring their β‐phosphoryl group onto phosphoserine residues. Several yeast proteins have been shown to be pyrophosphorylated *in vitro* (Saiardi *et al*., 2004; Bhandari *et al*., 2007; Wu *et al*., 2016), and two studies have implicated this non‐canonical post‐translational modification during intracellular vesicle trafficking (Azevedo *et al*., 2009; Chanduri *et al*., 2016). Protein pyrophosphorylation has the potential to affect the function of target proteins and directly transduce PP‐InsP metabolism into a signaling output; however, it has not yet been possible to directly identify pyrophosphorylated proteins from cell extracts. Advances in mass spectrometry methods (Penkert *et al*., 2017) are likely overcome this obstacle soon. Functional studies of non‐enzymatic processes are inherently problematic, but the fact that PP‐InsP‐mediated pyrophosphorylation requires pre‐phosphorylation of the serine residue (typically by casein kinase 2 and possibly other kinases) further hinders its *in vivo* characterization, since mutating the substrate serine would prevent the distinction of the kinase‐mediated phosphorylation and polyphosphorylation. An interesting observation in a recent study where synthetic pyrophosphopeptides were hydrolyzed after incubation with cell lysates suggests the presence of a protein pyro‐phosphatase (Yates & Fiedler, 2015). Identification of such a specific phosphatase may provide a good genetic tool with which to address pyrophosphorylation *in vivo*.

### III..3 Inositol pyrophosphate signaling in plants

**Figure 5:**
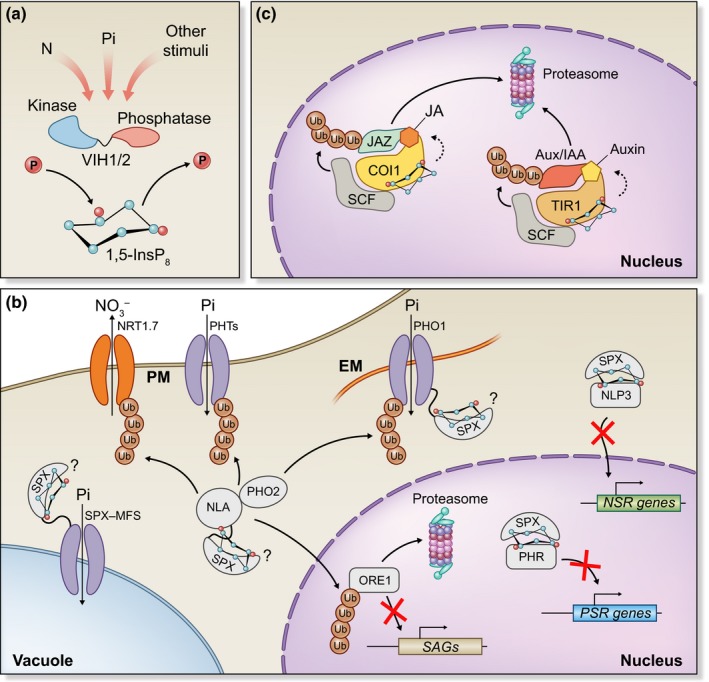
Speculative model of inositol pyrophosphate signaling in plants. Inositol pyrophosphates (PP‐InsPs) may control multiple aspects of plant signaling and physiology, namely responses to nutrient availability and hormone signaling. (a) PP‐InsP levels are expected to fluctuate according to nutrient availability, namely phosphate (Pi) and nitrogen (N), and to yet unidentified internal or external stimuli. Diphosphoinositol pentakisphosphate kinase (PPIP5K) enzymes (VIP1 HOMOLOG1/2 (VIH1/2) in *Arabidopsis thaliana*) can phosphorylate or dephosphorylate PP‐InsPs in reactions respectively catalyzed by their kinase or phosphatase domains; how the activities of each domain are balanced and how these enzymes integrate upstream signaling cues remains unknown. (b) PP‐InsPs are sensed by the SPX (SYG1/Pho81/XPR1) domains of multiple proteins. Stand‐alone SPX proteins bind to PHR transcription factors only in the presence of PP‐InsPs, preventing them from regulating the transcription of Pi‐starvation response (PSR) genes. In addition, SPX proteins prevent NODULE INCEPTION PROTEIN‐LIKE PROTEIN3 (NLP3) relocalization to the nucleus, where it regulates N‐starvation response (NSR) genes. The Ubiquitin (Ub) E2 conjugase and PHOSPHATE2 (PHO2) and the Ub E3 ligase NITROGEN LIMITATION ADAPTATION (NLA) ubiquitinate and regulate the accumulation of multiple target proteins, including PHOSPHATE1 (PHO1) and the PHOSPHATE TRANSPORTER (PHT) family of Pi‐transporters; the nitrate (NO_3_^−^) transporter NRT1.7; and the transcription factor ORESARA1 (ORE1), a key regulator of senescence‐associated genes (SAGs). The mechanistic details of PP‐InsP‐binding to NLA to SPX‐containing Pi‐transporters have not yet been investigated. (c) The auxin and jasmonic‐acid (JA) receptors use InsPs of PP‐InsPs as cofactors that facilitate ligand recognition and complex formation with their respective co‐receptors, Aux/IAA and JAZ proteins; these are then ubiquitinated by the TRANSPORT INHIBITOR RESPONSE1 (TIR1)/CORONATINE INSENSITIVE1 (COI1)‐Skp/Cullin/F‐box (SCF) complex and targeted for degradation, allowing auxin‐ and JA‐signaling activation.

As outlined above (section 3.2), PP‐InsPs control a wide range of cellular processes, many of which could also be present in plants. However, important roles for PP‐InsP in plants are only just emerging, and the contribution of PP‐InsPs for plant cell signaling and physiology requires investigation. Interestingly, the few examples of PP‐InsP‐mediated plant signaling events point to an involvement in plant‐specific processes and/or signaling cascades (Fig. 5).

#### PP‐InsPs and nutrient sensing

Eukaryotic SPX domains were recently described as high‐affinity PP‐InsP sensors (Wild *et al*., 2016). SPX‐containing proteins have long been associated with Pi sensing and metabolism in eukaryotes (Secco *et al*., 2012). SPX domains harbor a highly basic binding surface that shows great affinity to PP‐InsPs (KD values are in the micromolar to mid‐nanomolar range) (Wild *et al*., 2016), implicating PP‐InsPs in the regulation of SPX‐mediated Pi signaling. In fact, deletion of Kcs1 and/or Vip1 results in PP‐InsP depletion in yeast, and the mutant strains are impaired in several aspects of Pi signaling, including Pi acquisition, polyP synthesis and Pi starvation responses (Saiardi *et al*., 2004; Lee *et al*., 2007; Mulugu *et al*., 2007; Lonetti *et al*., 2011; Gerasimaite *et al*., 2017). Two Arabidopsis PPIP5K homologs, VIH1 and VIH2, are capable of suppressing yeast *vip1* null‐mutant phenotypes (Desai *et al*., 2014; Laha *et al*., 2015). Mutation of VIH2 in Arabidopsis, which shows a broader and stronger expression pattern compared to VIH1, eliminates production of InsP_8_ (Laha *et al*., 2015). Intriguingly, *vih2* mutant plants accumulate slightly more InsP_7_, a feature that is also observed in *vip1* yeast mutants (Norman *et al*., 2018). This most likely reflects an accumulation of the 5‐InsP_7_ isomer that is not further converted into 1,5‐InsP_8_, and implies that plants must encode for a yet unknown IP6K‐like enzyme capable of synthesizing this isoform. While single *vih1* and *vih2* Arabidopsis mutants exhibit rather mild developmental phenotypes, the *vih1 vih2* double mutant is seedling lethal (Zhu *et al*.,^2019^). By using this double mutant, it was possible to uncover an essential role for VIH1/2‐generated PP‐InsPs in plant phosphate starvation responses (Zhu *et al*.,^2019^). The *vih1 vih2* seedlings show constitutive high expression of Pi starvation marker genes and over‐accumulate Pi. The lethal phenotype is largely caused by over‐activation of Pi starvation responses (PSR), as a *vih1 vih2 phr1 phl1* quadruple mutant suppresses the *vih1 vih2* seedling phenotypes (Zhu *et al*., 2019). This reveals a critical role of VIH1/2‐derived PP‐InsP as negative regulators of PSR, and places their kinases genetically upstream of PHR1/PHL1. Depletion of PP‐InsP precursors should result in Pi‐related phenotypes similar to those of *vih1 vih2*. This is indeed the case for mutants affecting ITPK1 (phosphorylation of InsP_3_ and InsP_4_ on position 1) and IPK1 (conversion of InsP_5_ into InsP_6_), which also display phosphate over‐accumulation and developmental phenotypes (Kuo *et al*., 2018).

In the current working model (Fig. 5b), the PP‐InsPs produced by VIH1/2 during Pi‐sufficient conditions are sensed by stand‐alone SPX proteins, which can then sequester PHR1/PHL to prevent them from accessing their target promoters (Puga *et al*., 2014; Wang *et al*., 2014; Wild *et al*., 2016). Under Pi‐starvation, VIH1/2‐derived PP‐InsP concentrations are expected to drop. Without PP‐InsPs the SPX‐PHR1/PHL1 complex is no longer stable, and the transcription factors are set free to activate expression of Pi starvation genes. A similar mechanism is likely to have been adopted in other Pi‐dependent pathways, as OsSPX1/2 interact with the PHR1‐related RLI1 transcription factor to regulate Pi‐dependent leaf‐inclination in rice (Ruan *et al*., 2018).

PP‐InsPs are likely to be directly involved in the regulation of Pi transport in plants, as two Pi‐transporter families possess SPX domains: the SPX‐EXS and the SPX‐MFS subfamily of vacuolar Pi transporters (Secco *et al*., 2012) (Fig. 5b). Mutation of the PP‐InsP‐binding lysine cluster on the SPX domain of PHO1, the prototypical Arabidopsis SPX‐EXS, abrogates its ability to transport Pi from roots to shoots, causing a dramatic decrease in shoot Pi content (Wild *et al*., 2016). Similarly, mutations on the SPX domain of XPR1, a PHO1 homolog and the sole human SPX‐containing protein, are responsible for primary familial brain calcification disorders that are most likely caused by Pi transport deficiencies (Chande & Bergwitz, 2018). The mechanism by which PP‐InsP binding to the SPX domain activates these transporters has not yet been established.

It remains unclear which PP‐InsP species is the most prevalent during Pi signaling in plants. NMR experiments have confirmed that the Arabidopsis VIH2 synthesizes both 1‐InsP_7_ (from InsP_6_) and 1,5‐InsP_8_ (from 5‐InsP_7_) (Zhu *et al*., 2019). In yeast, the Pho80–Pho85–Pho81 CDK complex could be inhibited by cell extracts from *kcs1* but not *vip1* mutants (Lee *et al*., 2007), suggesting that 1‐InsP_7_ is sufficient for this action. However, *kcs1* mutants still exhibit deficiencies in Pi responses (Saiardi *et al*., 2004), indicating that other pathways may require different PP‐InsPs. This was demonstrated in the case of polyP synthesis in yeast, which is impaired in *kcs1* but not in *vip1* mutants *(Lonetti et al.,* 2011; Gerasimaite *et al*., 2017). Interestingly, 1‐InsP_7_ and 5‐InsP_7_ showed similar competence in terms of activating the VTC complex *in vitro*, while 1,5‐InsP_8_ was more effective (Gerasimaite *et al*., 2017). It is thus evident that yeast, and likely plants, specifically integrate distinct PP‐InsP isoforms to control Pi signaling, increasing its plasticity and complexity.

SPX domains have equally been implicated in nitrogen signalling, thus suggesting a broader role for PP‐InsPs in nutrient‐acquisition pathways. The rice NLP3, a key transcription factor for nitrogen responses, is controlled by OsSPX4, which is in turn ubiquitinated and degraded in a nitrogen‐dependent manner (Hu *et al*., 2019). Moreover, the Ub E3‐ligase NLA, which contains an N‐terminal SPX domain, controls nitrogen transport and nitrogen‐dependent senescence by regulating the Ub‐mediated degradation of nitrogen transporters and ORE1 (a key transcription factor for leaf senescence) (Peng *et al*., 2007; Liu *et al*., 2017; Park *et al*., 2018). Interestingly, NLA also controls the concentration of Pi transporters (Lin *et al*., 2013; Park *et al*., 2014; Yue *et al*., 2017), making it a hub that integrates signaling for both of these nutrients (Fig. 5b). How PP‐InsP affect these pathways has not been directly tested thus far.

In addition, PP‐InsPs have also been implicated in the regulation of target of rapamycin (TOR)‐mediated carbon metabolism in *Chlamydomonas* (Couso *et al*., 2016), and in the control of mitochondrial function and ATP homoeostasis in yeast and human cells (Gu *et al*., 2017a). How PP‐InsPs are able to integrate such a diversity of signaling pathways is far from understood. In yeast, contradictory results account for both increases and decreases in the concentrations of PP‐InsPs following Pi deprivation (Lee *et al*., 2007; Lonetti *et al*., 2011; Wild *et al*., 2016). Human cell lines showed a dramatic decrease in PP‐InsP concentrations that were rapidly restored upon Pi replenishment (Gu *et al*., 2017b). Interestingly, the same study showed an oscillation of cellular ATP concentrations in response to Pi availability. The mechanisms behind PP‐InsP fluctuation remain completely unknown, but it is tempting to speculate that cellular ATP concentrations may play a role, since yeast Vip1 and human PPIP5K2 kinase activities depend on high ATP concentrations (Gu *et al*., 2017b; Zhu *et al*., 2019). Moreover, since these enzymes exhibit both PP‐InsP kinase and phosphatase activity, it will be important to determine the contributions of each function to the maintenance of PP‐InsP concentrations (Fig. 5a).

#### Auxin and jasmonic acid signaling

The receptors for the phytohormones auxin and jasmonic acid (JA) – respectively TIR1 (and AFBs) and COI1 – share a common structure and mode of action. They are both leucine‐rich‐repeat (LRR)‐containing F‐box proteins that function as part of Skp1/Cullin/F‐box (SCF) ubiquitin E3 ligase complexes to promote ligand‐dependent degradation of their cognate co‐receptors and transcription repressors: Aux/IAA members for auxin, and the JAZ protein family in the case of JA (Pérez & Goossens, 2013) (Fig. 5c). TIR1 co‐crystallized with an InsP_6_ molecule derived from the insect cell expression host (Tan *et al*., 2007), and that mutation of InsP_6_‐binding residues disrupts the formation of a signaling‐active auxin‐TIR1‐AUX/IAA complex (Calderón Villalobos *et al*., 2012). This suggests that either InsP_6_ or a PP‐InsP acts as a co‐factor for TIR1, possibly facilitating auxin binding and subsequent co‐receptor recruitment. Although no auxin‐related phenotype has been reported thus far for VIH1/2‐depleted plants, genetic manipulation of the InsP pathway altered auxin signaling (Zhang *et al*., 2007, 2011; Chen & Xiong, 2010). This may, however, be caused by defects in PIP synthesis, InsP_3_ or calcium signaling, in addition to changes in PP‐InsP metabolism.

Similarly, the COI1‐JAZ‐JA/coronatin receptor complex was co‐crystallized in the presence of InsP_5_ (Sheard *et al*., 2010) and that mutation of COI1 InsP‐binding residues disrupts complex formation (Mosblech *et al*., 2011; Laha *et al*., 2016). Remarkably, InsP_6_ and especially InsP_7_ are more potent enablers of the assembly of the JA‐receptor complex than InsP_5_ (Laha *et al*., 2015, 2016). Computational modeling predicted InsP_8_ to be a more favorable ligand for COI1, inducing conformational changes that stabilize formation of the JA‐COI1‐JAZ receptor complex (Cui *et al*., 2018). Genetic disruption of VIH2 impaired JA‐dependent responses against herbivore larvae and necrotrophic pathogens (Laha *et al*., 2015). Since VIH2 synthesizes both 1‐InsP_7_ and 1,5‐InsP_8_, it remains unclear which PP‐InsP species is the preferred co‐factor for COI1. Consistently, Arabidopsis *ipk1* mutants (which fail to accumulate InsP_6_, while overaccumulating InsP_5_) have been found to be more susceptible to necrotrophic fungi, behaving similarly to *vih2* and *coi1* mutants (Stevenson‐Paulik *et al.,* 2005; Murphy *et al*., 2008; Laha *et al*., 2016). However, the same mutants were also reported to be hyper‐responsive to externally‐applied JA and more resistant to herbivores (Mosblech *et al*., 2011). Additional investigation is thus required to fully elucidate the roles of PP‐InsPs in JA signaling.

InsPs have been further implicated in plant immune signaling by the characterization of the *Xanthomonas campestris* effector protein XopH as phytase (InsP_6_ phosphatase) (Blüher *et al.,* 2017). Ectopic expression of XopH in *N. benthamiana* indeed resulted in reduced InsP_6_, InsP_7_ and InsP_8_ concentrations (Blüher *et al.,* 2017).

### III..4 Research tools and perspectives

Due to their low abundance and highly‐charged nature, detection and quantification of PP‐InsP concentrations *in vivo* still poses a major challenge. Available quantitative methods can distinguish the different isomers; however, these typically involve feeding cells/organisms with radio‐labeled PP‐InsP precursors (for a review, see Brown *et al*., 2016), followed by (bio)chemical extraction and chromatographic separation of the derived InsPs/PP‐InsPs. An accurate and systematic picture of the dynamics of PP‐InsPs under different conditions is still a distant vision. Nevertheless, a variety of tools has emerged that provide exciting research possibilities.

Most signaling mechanisms described so far require binding of PP‐InsPs to a given protein. Thus, identifying PP‐InsP‐interacting proteins is critical to uncover the pathways and processes governed by these molecules. The Fiedler lab at FMP Berlin devised an ingenuous protocol for the synthesis of a non‐hydrolyzable 5‐InsP_7_ analogue that carries a bisphosphonate group (PCP) in place of the usual pyrophosphate (Wu *et al*., 2016). This analogue was then linked to an affinity resin, allowing for protein pull‐down, much like a co‐immunoprecipitation, followed by mass spectrometry analysis to identify novel PP‐InsP‐interacting proteins in yeast (Wu *et al*., 2016). Similar approaches, using amine‐tethered or biotinylated InsPs, were employed to identify putative PP‐InsP binding proteins (Jiao *et al*., 2015; Gregory *et al*., 2016). These tagged InsP versions provide the added benefit of enabling attachment to a chip for SPR detection methods (Gregory *et al*., 2016), allowing for precise quantification of InsP–protein interactions that is critical to accurately distinguish PP‐InsP isomer‐binding preferences.

Genetic ablation of PP‐InsP‐producing enzymes often results in pleiotropic phenotypes and severe developmental defects that make it difficult to isolate specific processes or pathways. Delivering PP‐InsPs directly into live cells may be a good alternative; however, due to their polar nature they do not easily cross membranes. A membrane‐permeable photo‐caged version of InsP_3_ was engineered to allow UV pulse‐controlled InsP_3_ release in the cytosol, demonstrating that these molecules activate cytosolic calcium signaling within minutes (Li *et al*., 1998). More recently, a 5‐InsP_7_ photo‐caged analogue provided real‐time evidence for the translocation of the Akt PH‐domain from the plasma membrane to the cytoplasm (Pavlovic *et al*., 2016). These innovative approaches create exciting possibilities to study and visualize the effects of PP‐InsPs in single cells in a real‐time fashion.

Studying PP‐InsP‐mediated protein pyrophosphorylation presents perhaps the most demanding challenge. Without the possibility of genetically dissecting this process, one has to rely on *in vitro* biochemical methods; nevertheless, some sort of *in vivo* validation will be required to fully characterize pyrophosphorylation as a signaling mechanism. A promising new mass spectrometry technique was able to distinguish peptide pyrophosphorylation *in vitro* (Penkert *et al*., 2017) and may finally allow its detection from cell lysates. In addition, a fluorescent sensor designed to specifically bind diphosphate esters may provide agility in the identification and relative‐quantification of protein pyrophosphorylation (Williams & Fiedler, 2015).

A combination of these emerging new tools with traditional plant genetics and biochemical approaches may uncover the cellular functions of PP‐InsPs in plants and their underlying signaling mechanisms. Should the presence of polyPs be substantiated in higher plants, it will also be interesting to study the potential metabolic interactions between polyPs and PP‐InsPs.
